# *Scutellaria baicalensis Georgi* and Their Natural Flavonoid Compounds in the Treatment of Ovarian Cancer: A Review

**DOI:** 10.3390/molecules28135082

**Published:** 2023-06-29

**Authors:** Jiaying Cai, Qichao Hu, Zhelin He, Xiaoyan Chen, Jian Wang, Xiang Yin, Xiao Ma, Jinhao Zeng

**Affiliations:** 1 TCM Regulating Metabolic Diseases Key Laboratory of Sichuan Province, Hospital of Chengdu University of Traditional Chinese Medicine, Chengdu 610072, China; a13108078158@163.com; 2 Department of Gastroenterology, Hospital of Chengdu University of Traditional Chinese Medicine, Chengdu 610072, China; 3 School of Clinical Medicine, Chengdu University of Traditional Chinese Medicine, Chengdu 610075, China; 4 State Key Laboratory of Southwestern Chinese Medicine Resources, School of Pharmacy, Chengdu University of Traditional Chinese Medicine, Chengdu 611137, China; huqichaotcm@163.com; 5 Endoscopy Center, Guang’an Hospital of Traditional Chinese Medicine, Guang’an 638000, China; m18090520007@163.com (Z.H.); 13659096828@163.com (X.C.); 13568396305@163.com (J.W.); 18282619179@163.com (X.Y.)

**Keywords:** *Scutellaria baicalensis*, ovarian cancer, flavonoid, Chinese medicine, pathogenesis

## Abstract

Ovarian cancer (OC) is one of the most common types of cancer in women with a high mortality rate, and the treatment of OC is prone to high recurrence rates and side effects. *Scutellaria baicalensis* (SB) is a herbal medicine with good anti-cancer activity, and several studies have shown that SB and its flavonoids have some anti-OC properties. This paper elucidated the common pathogenesis of OC, including cell proliferation and cell cycle regulation, cell invasion and metastasis, apoptosis and autophagy, drug resistance and angiogenesis. The mechanisms of SB and its flavonoids, wogonin, baicalein, baicalin, Oroxylin A, and scutellarein, in the treatment of OC, are revealed, such as wogonin inhibits proliferation, induces apoptosis, inhibits invasion and metastasis, and increases the cytotoxicity of the drug. Baicalein also inhibits vascular endothelial growth factor (VEGF) expression etc. Analyzing their advantages and disadvantages in treating OC provides a new perspective on the role of SB and its flavonoids in OC treatment. It serves as a resource for future OC research and development.

## 1. Introduction

Ovarian cancer (OC) is one of the most common and deadly cancers in women worldwide, with the highest mortality rate and the worst prognosis of all gynecological malignancies. Many women are diagnosed with OC each year, and the 5-year recurrence rate for advanced OC can be as high as 70% [1,2]. Based on histological and embryological features, there are three main types of OC: epithelial, germ cell and mesenchymal gonadotrophic, of which the epithelial type is the most common, accounting for over 90%, with the last two types accounting for only about 5% of all ovarian cancers [3]. Since the fight against cancer began, the mortality rate from OC has decreased only slightly compared to other malignancies. The most effective treatment modalities for newly diagnosed OC are currently surgical treatment with primary surgical cytoreduction and chemotherapy with post-operative drugs. However, in patients with advanced OC, chemotherapy often leads to resistance to chemotherapy drugs, leading to cancer recurrence in more than 50% of patients [4,5]. In addition to drug resistance, the toxicity of chemotherapeutic drugs is often a challenge in the treatment of OC. In addition to drug resistance, the toxicity of chemotherapeutic drugs is often a challenge in the treatment of OC. The anticancer activity of chemotherapeutic drugs is proportional to their concentration in a dose-dependent manner, but larger doses of chemotherapeutic drugs often produce severe toxic side effects. For patients with OC, the toxic side effects of the drugs undoubtedly increase the burden on the body, and it is, therefore, crucial to find new ways to reduce drug resistance and toxicity.

*Scutellaria baicalensis* (SB) is a member of the family Labiatae and the genus Scutellaria, widely distributed in China, Russia, Mongolia, Korea, and Japan [6,7]. There are about 360 species in the genus Scutellaria, of which there are about 98 species in China. In China, SB has been widely studied and used since ancient times as a common traditional Chinese medicine and has been used medicinally for at least 2000 years. The stems and leaves of this plant are used to make tea for daily health care. Currently, many compounds have been found in SB. Most of these compounds are extracted from the roots of SB. The compounds can be classified into four structural types: free flavonoids, flavonoid glycosides, phenylethanoid glycosides and other small molecule compounds. Of these, the flavonoids and their glycosides are the main components, and according to some studies, these compounds can help fight cancerous diseases such as colon and liver cancer. In addition, the flavonoids of SB have various pharmacological activities, such as wogonin, which inhibits oxidative stress and regulates apoptosis, baicalein which inhibits cell growth; and baicalin which inhibits VEGF expression.

**Figure 1:**
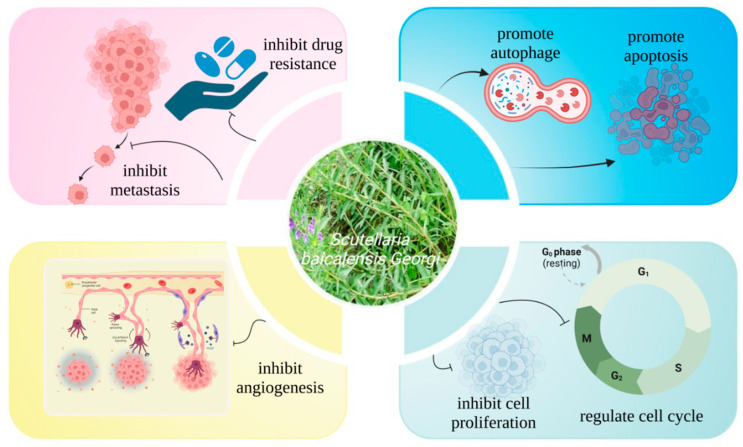
Inhibitory effect of *Scutellaria baicalensis* and its natural compounds on ovarian cancer. Created by Biorender.com.

With such a research base and the need for disease treatment, several studies have investigated the anti-OC properties of SB based on the anticancer effects of SB and its natural components (wogonin, baicalein, baicalin, Oroxylin A, Scutellarein). Therefore, this paper will review the pathological mechanisms of OC, elucidate the mechanisms of action of natural OC treatments and consider new approaches to the development of OC drugs (Figure 1).

## 2. Pathogenesis of OC

### 2.1. Cell Proliferation and Cell Cycle Regulation

Numerous studies have shown that the growth and proliferation of cells are closely related to the cell cycle. Cell proliferation, in turn, includes the replication of cytogenetic DNA and cell division, both governed by the cell cycle [8,9]. Studies have shown that the mammalian cell cycle consists of four phases: G1, S, G2 and M [10,11,12]. Cell cycle proteins, cell cycle protein-dependent kinases (CDK), and CDK inhibitors mutually regulate the four phases of the cell cycle [13].

As for cell cycle proteins, CDK6 and CDK4, together with D-type cyclins, form a complex for the activation of cellular mitogenic signaling, which has implications for the progression of cells towards the G1 phase. In numerous experiments, CDK6 was expressed at higher levels than CDK4 in OC [14,15,16,17]. High CDK6 expression predicts shorter progression-free survival in OC patients, while CDK4 overexpression rates are more variable, and CDK4 overexpression is closely related to p16, cyclin D1 [14,18,19,20]. Since CDK4 and CDK6 play essential roles in the cell cycle, many investigators currently conduct studies of CDK4/CDK6 inhibitors on OC. Zhang et al. evaluated the antitumour efficacy of a CDK4/6 inhibitor, abciximab, in a homozygous mouse OC model and observed that CDK4/6 inhibitors enhanced immune infiltration, the combination of CDK4/6i and anti-PD-1 antibodies improved the efficacy of anti-PD-1 therapy, which provides some direction for the treatment of OC [21]. Using different concentrations of fascaplysin, also a CDK4/CDK6 inhibitor, Chen et al. performed cell proliferation and cell cycle analyses on OC and found that fascaplysin induced S-phase block and apoptosis by measuring the levels of CDK4, cyclin D1 and Bcl-2. Fascaplysin treatment also inhibited the expression of CDK4, cyclin D1, Bcl-2 and VEGFA at the protein level [22].

CDK2 has a certain rate of expression in most ovarian tumours, both malignant and benign [23]. One study found that amplification of the cyclin E1 gene occurs in approximately 20% of high-grade plasmacytoma of the ovary and that Cyclin E1 induces entry into the S phase. This process is closely related to CDK2 [24]. Peng et al. studied Cyclin H in OC and found that Cyclin H was mildly expressed in grade 1 cancer tissues and highly described in grades 2 and 3. That additional silencing of the cell cycle protein H resulted in G1/S cell cycle arrest and inhibition of growth in OC cells [25].

### 2.2. Cell Invasion and Metastasis

Tumour invasion strategies include individual and collective invasion. Collective invasion is a fundamental characteristic of many metastatic tumours in human cancers, and the migration of multi-cellular clusters of OC cells in a directed and coordinated manner is collective invasion [26]. Tumour invasion is closely related to metastasis, which begins when cancer cells detach from the primary tumour and spread to surrounding tissues and distant organs. This process occurs in approximately 90% of cancer-related deaths [27,28]. In OC, tumours metastasize by abdominal metastases versus blood metastases. Abdominal metastases, which migrate directly through the peritoneal fluid and spread within the abdominal cavity, are the most common route of metastasis in OC and often affect other vital organs in the abdominal cavity [29,30]. The incidence of peritoneal and abdominal metastases is relatively higher than that of extra-abdominal metastases. Therefore blood metastases are considered to play a minor role in the metastatic process of OC [30]. Various signaling molecules and pathways are essential in OC cell invasion and migration. Therefore, several signaling pathways closely related to OC will be described here.

#### 2.2.1. PI3K/AKT Signaling Pathway

34% of the samples examined showed alterations in the PI3K/AKT/mTOR signaling pathway, particularly frequent in OC [31]. The regulation of cell growth, proliferation, transcription, translation, and metabolism depends heavily on this signaling system [32,33,34].

The PI3K/AKT/mTOR signaling pathway’s mode of action in OC is complicated. Its abnormalities, however, are connected to the development of tumors and the invasive spread of cancer cells [35,36]. PI3Ks are lipid kinases, and the phosphatase and tensin homolog (PTEN) deficiency in ovarian surface epithelial cells causes tumorigenesis dependent on the p110β isoform of PI3K [37]. A functional shortage of PTEN reduces its lipid phosphatase activity, which is necessary for tumor suppressive action and is thought to be a critical negative regulator of PI3K signaling [38,39]. The PI3K/AKT/mTOR signaling pathways of siRNA-targeted elements have also been connected to OC. It was demonstrated that p110 siRNA-transfected OC cells showed decreased proliferative and migratory potential and cell cycle arrest [40]. It has also been demonstrated that blocking mTORC1 and mTORC2 prevents OC cells from proliferating and migrating [41,42,43].

Outside variables also have a role in the PI3K/AKT/mTOR signaling pathway’s aberrant activation in OC, in addition to the signaling molecules’ impact. In most OC cases, epidermal growth factor receptor overexpression was discovered [44]. AKT is one of the downstream targets of epidermal growth factor (EGFR) and is activated by phosphorylation at the Ser-473 site. In the emergence and evolution of OC, miRNAs play two distinct roles. On the one hand, miRNA-182 encourages OC cell growth [45], and miR-204-5p promotes ovarian tumor angiogenesis [46]; on the other, miRNA-204 expression deletion encourages OC cell migration and invasion [47].

#### 2.2.2. NF-κB Signaling Pathway

An NF-κB signaling pathway is one of the essential signaling pathways in OC pathogenesis, including classical and non-classical NF-κB signaling pathways. Pathway activation promotes chemoresistance, invasion, migration and immune evasion.

In OC cells, inhibitory kappa beta kinase β (IKKβ) promotes OC growth, proliferation and invasion, while the p65 gene promotes the spherical growth of OC cells [48,49,50,51]. phosphorylation of IKKβ and p65 activates NF-κB and increases TRIM52 expression. TRIM genes are associated with upstream regulation of the NF-κB pathway. Yang et al. further demonstrated that the knockdown of TRIM52 inhibited OC cell invasion and migration and induced apoptosis [52]. Lídia Hernandez et al. validated the effect of IKKβ signaling in OC-on-OC cell proliferation, invasion, and adhesion. Also, they blocked IKKβ interference through small molecule inhibition, and the structure reduced the invasiveness of most OC cell lines. In addition, the deletion of RelB reduced metastasis of OC tumours to abdominal organs [48].

#### 2.2.3. Wnt/β-Catenin Signaling Pathway

Activation of the Wnt/β-catenin signaling pathway, like activation of the NF-κB signaling pathway, can promote cancer cell self-renewal, tumor angiogenesis, and tumor immunity. However, it supports epithelial-to-mesenchymal transition (EMT) [53]. E-cadherin and β-catenin activities, for example, are essential in initiating EMT in OC cells. And E-cadherin reduction would promote the β-catenin signaling pathway [54,55].

When Wnt ligands bind to Fzd/LRP6/LRP5, Dsh phosphorylation triggers the typical Wnt signaling pathway, which controls cell proliferation, differentiation, and secretion. Studies have shown that Wnt ligands, Fzd1, Fzd5, LRP5/6, and β-catenin proteins in the Wnt/β-catenin signaling pathway are significantly upregulated in OC and that this pathway is involved in the regulation of follicular development [2,56]. For instance, Barghout et al. discovered that down-regulating β-catenin expression increased the sensitivity of cancer cells to chemotherapeutic drugs. In contrast, up-regulating β-catenin activity caused OC cells to become resistant to carboplatin. Many medication development concepts have focused on decreasing β-catenin activity [54,57]. According to Song et al., miR-219-5p inhibited Wnt/β-catenin signaling and autophagy by targeting high mobility group AT-hook 2 (HMGA2) [58]. Ruan et al. revealed that Leucine-rich-repeat-containing G protein-coupled receptors (LGRs) downregulation inhibited the Wnt/β-catenin signaling pathway and enhanced the sensitivity of OC cells to chemotherapy [59]. These studies all showed that blocking or deactivating the Wnt/β-catenin signaling pathway prevented the growth of OC cells.

#### 2.2.4. MET/HGF Signaling Pathway

c-MET regulates cell signaling and cytoskeletal rearrangement, and studies have shown that c-MET is overexpressed in 7% to 27% of OCs [60,61]. Intraperitoneal treatment with MET siRNA significantly reduces tumour burden, ascites formation, protease activity and peritoneal implants [62]. Evidence shows that MET is induced under hypoxic conditions, impeding cancer therapy, and promoting metastasis. MET upregulation sensitizes cells to HGF, activating an invasive growth program leading to cell migration and extracellular matrix (ECM) invasion [63]. HGF affects the peritoneal adhesion of OC cells, a key OC metastasis mechanism that induces matrix metalloproteinases (MMPs) activity at multiple levels [64]. Various studies have shown that increased tumour aggressiveness is associated with the upregulation of MMP9 [65,66]. Davies et al. inhibited ovarian tumour invasion by MMP inhibitors, further demonstrating that the protease affects tumour aggressiveness [67].

#### 2.2.5. MAPK Signaling Pathway

The MAPK signaling pathway is one of the more well-studied pathways in cancer biology, and its over-activation causes more than 40% of cancers. There are three significant subfamilies of MAPK: MAPK/ERK, Ras/Raf1/MEK/ERK; JNK or SAPK; and MAPK14. Within these three subfamilies, in turn, there exists a large number of ERK-MAPK studies [68]. In OC, activation of the MAPK/ERK pathway leads to increased proliferation and invasiveness of OC cells [69]. For example, RAY et al. identified a human-derived IgM monoclonal antibody, Mab216, that binds to poly-N-acetyl lactosamine in blood and solid tumours, leading to MAPK pathway inactivation and thus reduced OC cell invasiveness [70].

### 2.3. Drug Resistance

In chemotherapy for OC, commonly used drugs include platinum-based drugs such as cisplatin and carboplatin, taxanes such as paclitaxel and doxorubicin, anti-angiogenic drugs such as bevacizumab and trebananib, and other drugs such as pegylated liposomal doxorubicin and gemcitabine, which are often used in combination [71]. However, over 70% of patients relapse after treatment and eventually become drug resistance [72]. Drug resistance often includes both intrinsic and acquired resistance. Intrinsic resistance mechanisms include drug degradation by drug-metabolizing enzymes, mutations in drug targets and alterations in drug membrane transport [73,74]. Whereas acquired drug resistance is often associated with overexpression of drug efflux proteins [75,76]. Drug efflux proteins are an important link to drug resistance in OC, exemplified by ABCB1. ABCB1 reduces the concentration of chemotherapeutic drugs in OC cells [77]. Shan et al. found that adriamycin upregulated the expression of CCL20 in SKOV3, and CCL20 enhanced the resistance of OC cells to adriamycin by regulating the expression of ABCB1 [78].

### 2.4. Angiogenesis 

Angiogenesis, the process by which luminal endothelial cells migrate through the vascular basement membrane to the underlying extracellular matrix to form elongated germinal forms, is the main mechanism of angiogenesis [79]. Tumour growth and metastasis depend on the growth of blood vessels within the tumour, and the VEGF family is one of the most important regulatory molecules in normal ovarian angiogenesis and tumour-induced angiogenesis [80]. There are four human VEGFs in the VEGF family, namely VEGF-A to -D, which bind to the three VEGF receptors on the vascular endothelium [81]. In contrast, VEGF-A has been most studied in OC. VEGF expression continues to increase as the tumour progresses. In Zhong et al.’s study, VEGF overexpression was found to reverse the inhibitory effect of FBXW7 overexpression on OC cell invasion, migration and angiogenesis, and the expression levels of VEGFR1 and VEGFR2 were found to be upregulated [82]. Therefore, some studies have found that inhibition of angiogenesis could be a direction for treating OC [83,84]. For example, poplar leaf proanthocyanidins showed a strong growth inhibitory effect on cisplatin-resistant A2780/CP70 cells by inhibiting angiogenesis [85]. 

### 2.5. Apoptosis and Autophagy

#### 2.5.1. Apoptosis

Apoptosis is a programmed cell death that usually involves two mechanisms: an exogenous pathway and an intrinsic pathway [86,87,88]. Cell surface receptors’ interaction with TNF superfamily-related protein ligands starts the exogenous route [89]. The intrinsic apoptotic pathway is mainly associated with the BCL-2 family and IAP [90,91,92]. Studies have shown that cancer development is associated with abnormalities in both pathways. 

The Bcl-2 family was discovered to be overexpressed in OC cell lines [93,94]. And the Bcl-2 family has both pro-apoptotic and anti-apoptotic members, such as Bax, Bok, and Bcl-2, Bcl-XL [93,95,96]. A study detecting Bcl-2 in OC cell lines found that Bcl-2 expression was relatively low in OC-resistant cell lines but high in sensitive cell lines [97]. Dai et al. also found that Bcl-2 could promote OC cell survival and drug resistance in these cells [98]. The IAP family, which includes XIAP and Survivin, is another factor that controls intracellular apoptosis. And XIAP can control apoptosis by blocking Caspase-3, Caspase-7, and Caspase-9 [99,100,101]. In addition to this, it also transmits its apoptotic effects through the cleavage of Caspase-3 and PARP in OC cells [102]. And Survivin blocks apoptosis by inhibiting Caspase-3 and Caspase-7 [103]. 

#### 2.5.2. Autophagy

Autophagy is a metabolic pathway that degrades damaged, old, non-essential or abnormal molecules and organelles. The process consists of three main steps: molecules and organelles enter the autophagosome through LC3 recruitment; lysosomes merge with autophagosomes to form autophagic lysosomes; and lysosomal enzymes degrade vesicle contents, which are then reused by cells. Studies have revealed that autophagy is crucial for developing cancer, autoimmune diseases, and allergies [104,105]. 

**Figure 2:**
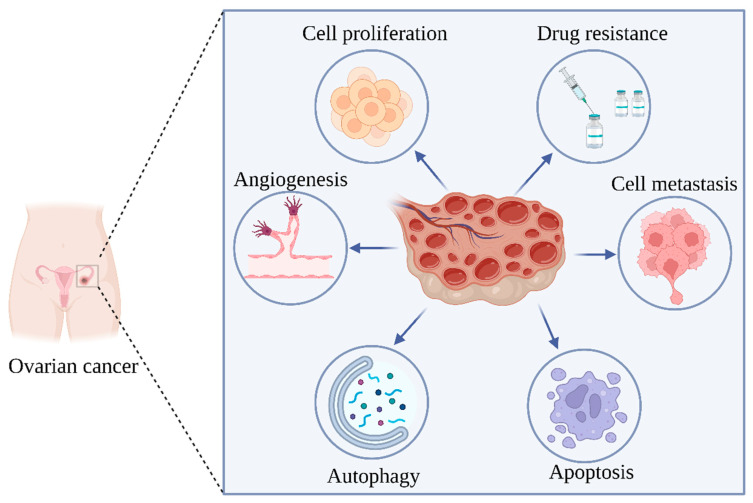
Pathogenesis of ovarian cancer. Created by Biorender.com.

According to a number of studies, the process of autophagy has a dual function in the emergence of OC. On the one hand, inhibition of autophagy promotes ovarian tumor survival. According to Liang et al.’s research, Beclin 1, a gene that promotes autophagy, has a single allele deletion in 40–75 percent of OCs [106]. Then another study showed that patients with high levels of Beclin-1 had a better prognosis than patients with low levels [107]. Autophagy can also contribute to cancer cell survival by providing oncogenic metabolites. Autophagy releases IL-6 from CAFs and promotes OC cell migration [108]. For instance, under hypoxia conditions, OC cells exhibit increased levels of LC3A SLS expression, which suggests that autophagy is enhanced in these cells to combat hypoxic stress and lengthen survival [109]. The findings above imply that changes in autophagy can affect the growth of OC tumors (Figure 2). 

## 3. Inhibitory Effects of the Natural Ingredients on OC

### 3.1. Scutellaria Baicalensis Extract

Some studies found that SB extract could inhibit cell invasion and regulate the cancer cell cycle. For example, treatment of OC cells with SB reduced the activation of NF-κB, inhibited the growth and invasion of cancer cells, down-regulated CXCR4 and MMP-9 in OC cells, and reduced the invasive potential of ovarian and endometrial cancer cells [110]. Besides, SB extract also activated caspase-3, caused G0/G1 phase cell cycle arrest, down-regulated cell cycle protein d1 and d3 expression, and up-regulated p27 expression, which affected the cancer cell cycle [111]. Besides that, SB extract induces apoptosis and enhances the cytotoxic effect of cisplatin. Using when 400 μg/mL SB extract and 30 μM cisplatin were simultaneously applied to sensitive and resistant cells, Chol et al. found that SB extract induced apoptosis by inducing the expression of p53, p21, Bax, Atg and Atg12 in the cisplatin-sensitive OC cell line A2780 [112]. SB extract also accelerated HIF-1α degradation through proteasomal and lysosomal pathways, PI3K/AKT and MEK/ERK pathways inhibited HIF-1α expression, reduced ABCG efflux proteins and enhanced cisplatin cytotoxic effects. Compared with baicalin extract alone, cisplatin-sensitive OC cells and cisplatin-resistant cells responded similarly to baicalin extract and cisplatin, and the results indicated that the combination of baicalin extract and cisplatin had a synergistic effect on cisplatin-resistant cells [111,112].

### 3.2. Wogonin

Wogonin (5,7-dihydroxy-8-methoxy-2-phenylchromen-4-one) is the main bioactive component of SB, which has the chemical formula C16H12O5 and molecular weight of 284.26 g/mol and is a dihydroxy and monomethoxyflavone. It is generally extracted from the roots of the plant. Modern pharmacological studies have found that aconitine has a wide range of pharmacological activities, such as antiviral, antioxidant, antibacterial, anticancer, anxiolytic and neuroprotective effects, and is used in the treatment of anemia, hypertension, hyperlipidemia, atherosclerosis, rheumatism, fever, bacterial and infectious diseases [113].

In OC, wogonin mainly inhibits cell growth and promotes apoptosis. The results of Ruibin et al.’s research showed that treatment with wogonin inhibited the proliferation and reduced the invasiveness of A2780 cells and that the degree of inhibition correlated with the concentration of the drug. MPP is a specific ER inhibitor, and wogonin combined with MPP reduced the expression of cyclin D1, CDK4, and CDK6 with the percentage of G0/G1, which significantly enhanced the antitumor effects of baicalin in A2780 cells [114]. Zhao et al. reported that wogonin could upregulate p53 and TIGAR and downregulate PGM, HK2, GLUT1, PDK and LDHA. This suggested that it prevented the transplanted tumor A2780 xenografts from growing and metabolizing glycolysis, which relies on wt-p53 [115]. In a study by Zhao et al., baicalin has cytotoxic effects on OC cells since it inhibits the growth of Caov-3 and A2780 cells at a 200 μM concentration and induces apoptosis in A2780 via raising the Bax/Bcl-2 ratio [116]. Chen et al. detected p53 and Akt in A2780 and PTX10 cell lines. They showed that baicalin down-regulated the expression of the Akt protein in PTX10 and up-regulated the expression of p53 in A2780, suggesting that baicalin could promote apoptosis [117].

In OC, wogonin also enhances the cytotoxic effect of cisplatin. Yang et al. claim that wogonin decreases catalase activity and raises intracellular hydrogen peroxide, which improves the susceptibility of OC cells to tumor necrosis factor-induced apoptosis. They discovered that SKOV3 had synergistic cytotoxicity when 20 μM baicalin and various dosages of TNF were employed. The researchers also showed a ROS-dependent mechanism whereby baicalein inhibited NF-κB activation [118]. Xing et al. found that for SKOV3/DDP, the percentage of apoptotic cells was higher in the baicalein plus cisplatin group than in the baicalein and cisplatin alone group. In addition, they investigated the PI3K/Akt pathway and showed that baicalein increased the sensitivity of cisplatin by inhibiting the PI3K/Akt pathway [119]. Wogonin affects angiogenesis in OC. Wogonin inhibits the activity of Indoleamine 3,5-dioxygenase-1 in A278 cells, thereby improving the immunosuppressive environment of cancer and attenuating the metastatic potential. In vitro and mouse models, wogonin can down-regulate the expression of VEGF genes or proteins in various cancer cells, thereby reducing angiogenesis and cancer cell migration [120]. 

### 3.3. Baicalein

Baicalein (5,6,7-trihydroxy-2-phenyl-4H-chroMen-4-one) is a phosphate compound extracted from SB root. Its chemical formula is C15H10O5, with a molecular mass of 270.24 g/mol, and is a trichophenolone based on C-5, 6- and 7-bits. It is therapeutic against bacteria, inflammation, cancer, viruses, and allergies. Its anti-cancer effects include suppressing cell proliferation-inducing and autophagy cell death [8]. A study showed that baicalein inhibited the viability and proliferation of cancer cells while having a weaker inhibitory effect on normal cells, which would help reduce the toxic effects of cancer treatment. Baicalein significantly inhibited HIF-1α expression at 20 μM and 40 μM concentrations and also had inhibitory effects on the expression of c-Myc, NF-κB and other pro-oncogenes. The inhibitory effect on vascular endothelial growth factor was enhanced with the increase in treatment concentration [121]. In a study of the anticancer effects of baicalein on A2780, SKOV3 and OVCAR cell lines, Pan et al. observed that baicalein inhibited the viability of these three cell types and suppressed A2780 cell proliferation through the Akt/β-catenin signaling pathway. In addition to inhibiting cell proliferation, Baicalein also induced apoptosis. Baicalein induced apoptosis in A2780 cells through the intrinsic apoptotic pathway and increased caspase-3 and PARP activity with paclitaxel to promote apoptosis in human OC cells [122]. Wang et al. found that combined treatment with chloroquine and baicalein significantly reduced cell viability and increased PARP cleavage in HEY and A2780 cells, whereas treatment of HEY and A2780 cells with different concentrations of baicalein alone induced Beclin 1 and ERK-dependent autophagy in ovarian HEY cancer cells. In addition, they found that phosphorylation of ERK, Thr202/Thr204, and AKT increased after baicalein treatment. These results suggest that baicalein inhibits HEY cell proliferation and induces apoptosis [123].

Baicalein also inhibits cell invasion through NF-κB signaling and suppresses VEGF expression. Yan et al. showed that baicalein reduced the expression of MMP-2 and significantly inhibited the invasion of OC cells. They also found that baicalein reduced the activation of NF-κB signaling molecules and inhibited the activation of p38. In the presence of pyrrolidine dithiocarbamate (PDTC) combined with baicalin, the expression and invasion of MMP-2 protein in OC cells were significantly inhibited through NF-κB signaling. In conclusion, the anti-metastatic properties of baicalein were exerted through the inhibition of MMP-2 expression and invasion of OC cells [124]. In a study of biologically active phenolic compounds, He et al. found that baicalein had a moderate inhibitory effect on protein expression of vascular endothelial cell growth factor, with the strongest inhibition of OVCAR-3 and A2780/CP70 proliferation at 40 μM [125]. 

### 3.4. Baicalin

Baicalin (7-D-Glucuronic acid-5,6-dihydroxyflavone) is a glycosidic flavonoid, a 7-O-glucuronide in baicalin, which has the chemical formula C21H18O11 and a molecular weight of 446.4 g/mol. It is present in various plant families, including Lignanaceae and Labiatae, and is mainly isolated from different species of Scutellaria, with antiproliferative potential and modulation of various signaling pathways [126]. Baicalin has been shown to suppress OC cell growth, induce apoptosis in OC cells and inhibit the expression of VEGF. Treatment with 15 μM baicalin for 24 h significantly inhibited spherical growth, spheroid formation and tumor initiation frequency of OC cells by Li et al. In addition, non-toxic doses of baicalin treatment inhibited the expression of OC stem cell markers. The results showed that baicalin inhibited stem cell inhibition in OC cells by attenuating Yes-associated protein (YAP) activity through inhibition of RASF6 at the transcriptional level [127]. In research by Gao et al., cell vitality was assessed using the MTT assay. Baicalin treatment drastically decreased the viability of cancer cells when compared to untreated cells. However, it did not affect normal ovarian cells, demonstrating its safety. 39.5% of apoptotic cells were found in the 160 μM baicalin-treated group, proving that baicalin caused apoptosis in A2780 OC cells [112]. He et al. used 40 μM to act on two OC cell lines for 24 h. The results showed that baicalin inhibited VEGF expression in OC cell lines like cisplatin [125]. 

### 3.5. Oroxylin A

Oroxylin A (5,7-dihydroxy-6-methoxy-2-phenylchromen-4-one) is a monomethoxyflavone and a dihydroxyflavone, where the hydroxyl group is located at C-5 and C-7, and the methoxy group is located at C-6. Its chemical formula is C16H12O5, and its molecular weight is 284.26 g/mol. has an antitumor agent and EC 1.14.13.39 (nitric oxide synthase) inhibitor effects. It is a flavonoid derived from SB, Oroxylum indicum and other plants that modulates PTEN/PI3K/Akt, NF-κB, MAPK, Wnt/β-catenin and other signaling pathways [128]. In OC, Oroxylin A has been found to play a role in two main areas. The first effect is to enhance the cytotoxic effect of cisplatin. In NCI/ADR-RES cell lines, Oroxylin A successfully lowered P-glycoprotein-mediated drug efflux, improving the sensitivity of chemotherapeutic drugs to cancer cells. Go et al. looked at how Oroxylin A affected the cytotoxicity of vincristine and paclitaxel, and they discovered that doing so increased sensitivity to the cytotoxicity of both drugs [129]. Another effect is the induction of apoptosis in OC cells. Mu et al. found that Oroxylin A inhibition of cancer cell viability was associated with p53 status, which -induced apoptosis in OC cells by upregulating p53 [130]. According to Shen et al., Oroxylin A altered the expression profile of PGRMC1/2, reduced PPAR expression at high dosages, and further triggered apoptosis in SKOV-3 OC cells [131]. 

### 3.6. Scutellarein

**Figure 3:**
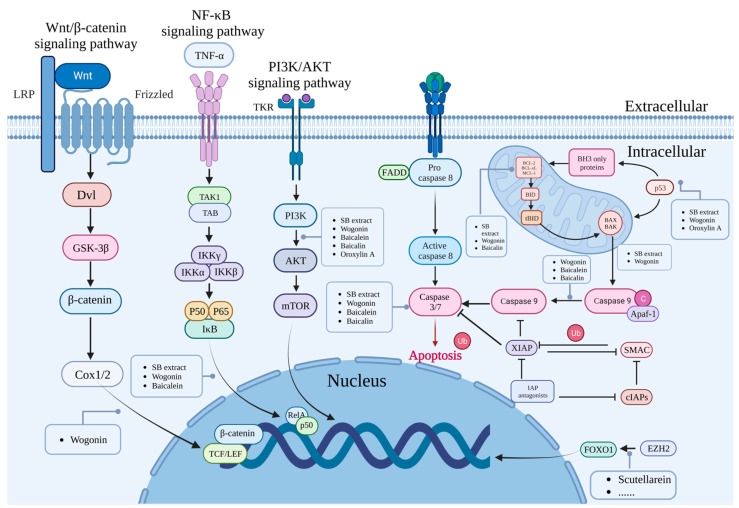
Anti-ovarian cancer mechanism of *Scutellaria baicalensis* and its natural compounds. Created by Biorender.com.

Scutellarein (5,6,7,4′-Tetrahydroxyflavone) is another flavonoid substituted with hydroxyl groups at the C-4′, -5, -6 and -7 positions. It has the chemical formula C15H10O6 and a molecular weight of 286.24 g/mol, and exhibits anticancer effects in many human cancers [132]. Lang et al. found that in A2780 and SKOV-3 OC cell lines, treatment with scutellarein reduced their proliferation rates, with maximum growth reduction occurring after 48 h at 100 μM concentration, inhibiting the migration and invasion of cancer cells. In addition, EZH2 inhibited FOXO1 expression by inducing H3K27me3 in OC cells, reducing EZH2 expression in OC cells [133] (Figure 3). 

## 4. Pharmacokinetic Data

Moreover, SB and its natural flavonoid constituents have certain advantages in pharmacokinetics. There have been numerous studies on the pharmacokinetics and pharmacodynamics of SB and its natural flavonoid constituents. SB is included in Huangqin Tang, and studies have shown that the distribution, absorption, and elimination of the drug are delayed after administration, thereby prolonging the metabolism of the drug in the body. Baicalein, baicalein, and baicalein were still detectable in urine 36 h after administration. It also exhibits increased peak concentrations and area under the concentration-time curve and is more effective in exerting therapeutic effects [134]. Pharmacokinetic studies of Wogonin have shown that intragastric administration of Wogonin results in poor absolute bioavailability of 1.10%. Wogonin had a quick dispersion but noticeably larger amounts in the liver and kidneys after intravenous administration [135]. Another study found that baicalin had the highest concentration in the kidneys of rabbits after animal administration, while the highest concentration of baicalin was detected in the lungs of rats, followed by the kidneys and liver [136]. Baicalein and baicalin are excreted in feces mainly through biliary excretion as glucuronide and sulfate, with minimal urinary excretion [137]. Oroxylin A is widely distributed in the liver and can also occur in the nuclei of tumor cells; its metabolites are more widely distributed in the kidney [138,139]. Rat brain and plasma have both been discovered to contain Oroxylin A, indicating they can pass the blood-brain barrier [140]. These results, in part, imply that SB and its flavonoids can be transported to various tissues and organs and have various pharmacological effects after entering the body.

## 5. Advantages and Disadvantages of Compounds

In addition to *Scutellaria baicalensis* and its natural flavonoids, which have anticancer activity against ovarian cancer, several other natural products can also produce similar effects. Curcumin, mainly from turmeric root, has been found in studies to inhibit the proliferation and metastasis of ovarian cancer cells, and it causes cell cycle arrest in the G2/M phase and leads to cell death. Resveratrol, a polyphenolic compound, and ginsenoside, a steroidal compound, both have anticancer activity similar to curcumin [141]. Compared to these natural products, SB and its natural compounds have an advantage in the fight against OC. The benefits are mainly in two areas, anticancer effect and pharmacokinetics. On the one hand, baicalin enhances the anticancer effects of cisplatin, attenuates cisplatin-induced cachexia and acute kidney injury [142], and enhances the cytotoxic effects of cisplatin on cisplatin-sensitive and resistant OC cells by inhibiting MAPK/ERK and PI3K/AKT pathways [111,112]. Baicalein and baicalin significantly impact the survival and proliferation of OC cells yet have less inhibitory effects on normal cells. Low concentrations of wogonin were relatively safer and showed no significant toxicity in experimental studies. Oroxylin A showed a significant reduction of oxidative stress and enhancement of antioxidant enzyme activity in different models [143]. Therefore, these substances have some anticancer potential. However, further clinical studies are necessary to investigate the optimal dose of these substances in humans and their efficacy and safety. 

There is abundant evidence that SB and its flavonoids significantly inhibit tumour growth in both in vitro and animal studies. However, few clinical trials have confirmed their efficacy in cancer treatment and chemoprevention. To date, the effects of baicalein on cancer and normal cells and its mechanisms are not fully understood, the targets of action are unclear, and further research into targets for cancer therapy is needed. In addition, baicalein and baicalin are still difficult to use in clinical applications due to their low bioavailability, poor solubility and rapid metabolism.

## 6. Conclusions

This paper reviews the common pathogenesis of OC, including cell proliferation and cell cycle regulation, cell invasion and metastasis, drug resistance, apoptosis and autophagy, angiogenesis and other aspects, summarizes the research application of SB and its flavonoids Wogonin, Baicalein, Baicalin, Oroxylin A, and Scutellarein in OC and their antioxidant mechanisms. The research application of SB and its flavonoid compounds in OC and their anti-OC mechanism were compared, and the advantages and shortcomings of SB and its natural flavonoid compounds in the treatment of OC were investigated (Table 1).

This review found that wogonin, baicalein, and baicalin are more studied in the treatment of OC, which may be related to their higher content. Among these flavonoids, wogonin and baicalein have more pharmacological effects, inhibiting cell growth, inhibiting apoptosis, inhibiting angiogenesis, inhibiting tumour invasion and enhancing the cytotoxicity of the drugs. Interestingly, some studies have shown that baicalein has a faster and more potent anticancer effect than baicalin. Similarly, baicalein has been shown to have the strongest inhibitory effect in several studies looking at flavonoids. This may be due to the higher number of hydroxyl groups on the baicalein A ring and the smaller size and higher lipophilicity of baicalein elements [125,146]. Both baicalein and baicalin significantly inhibited the expression of HIF-1α, c-Myc, NF-κB, VEGF and others. However, baicalein was more effective in inhibiting cancer cell proliferation and HIF-1α, c-Myc, NF-κB and VEGF expression [121]. In addition to the natural components of baicalein, derivatives of the natural components can also be used in cancer treatment. For example, additionally to wogonin, its derivatives are employed in treating cancer. GL-V9 has anti-cancer solid action, while compound 51 is selective for CDK9 overexpressing cancer cells and suppresses the proliferation of MV4-11. LW-213 inhibits the Akt/Gsk3/catenin signaling system to produce anticancer effects on cell proliferation and the cell cycle. In a study on the treatment of OC, it was discovered that the wogonin derivative FV-429 bound to HIF-1 and improved paclitaxel resistance reversal through G2/M phase blockade while inhibiting c-Src and blocking the c-Src/STAT3/HIF-1 pathway. This has significance for treating OC by paclitaxel resistance reduction [144].

In conclusion, from the current research, SB and its flavonoids have good application prospects in treating OC. It provides ideas for developing therapeutic drugs for OC, and future research may find new targets and methods for treating OC.

## Figures and Tables

**Table 1:** Profiles of SB and its flavonoid compounds in OC therapy.

Compounds	Cell Lines	Dosage	Mechanism of Action	References
Wogonin	SKOV3	0–160 μM (In vitro)	↓p-Akt	[119]
	A2780	19.9 ± 1.2 μM (In vitro)	/	[120]
	A2780	0.4–12.7 μg/mL (In vitro)	↓Akt, ↑p53	[117]
	SKOV3	5–20 μM (In vitro)	↓c-Src, ↓STAT-3, ↓HIF-1α	[144]
	A2780	0–50 μM (In vitro)	↓ER-α, ↓VEGF, ↓Bcl-2, ↓Akt, ↑Bax, ↑p53	[114]
	A2780	0–80 mg/kg (In vitro)	↓MDM2, ↓glycolysis, ↑p53	[115]
	A2780	20–80 mg/kg (In vivo)	↓glycolysis, ↑p53	[115]
	A2780	0–200 μM	↑Bax, ↓Bcl-2	[116]
	SKOV3	0–20 μM	↓ROS, ↓NF-κB	[118]
Baicalein	HEY, A2780	12.5–50 μM	↑LC3-II, ↑ERK, ↑Akt	[123]
	OVCAR-3, A2780/CP-70	20, 40 μM	↓VEGF	[125]
	A2780, SKOV3, OVCAR	1–1000 μM	↑caspase-3, PARP	[122]
	SKOV3, CAOV3	0–12.5 μM	↓MMP-2, ↓NF-κB, ↓p38	[124]
	OVCAR-3, CP-70	0–160 μM (In vitro)	↓VEGF, ↓HIF-1α, ↓c-Myc, ↓NF-κB	[121]
Baicalin	OVCAR-3, A2780/CP-70	20, 40 μM (In vitro)	↓VEGF	[125]
	A2780, A2780cis	28 μM, 56 μM (In vitro)	Reduced the cell proliferation	[112]
	A2780	40–240 μM	↓MMP-2, ↓MMP-9, ↓Bcl2, ↑caspase-3 and -9	[145]
	OVCAR-3	15 μM, 40 μM	↓CD133, ↓ALDH1A1, ↓YAP	[127]
	OVCAR-3, CP-70	0–160 μM (In vitro)	↓VEGF, ↓c-Myc, ↓NF-κB	[121]
Oroxylin A	NCI/ADR-RES	0.01–40 μM	↓P-gp	[129]
	SKOV-3	≥200 μM	/	[130]
	SKOV-3	20–800 μM	↑PPARγ	[131]
Scutellarein	A2780, SKOV-3	25–100 μM	↓EZH2, ↑FOXO1	[133]
SB extract	SKOV3, OVCA-429, OVCA-420	100 μg/mL	↓HIF-1α, ↓ABCG1, ↓ABCG2	[111]
	A2780, A2780cis	0–400 μM	↑p53, ↑p21, ↑Bax, ↑Atg5, ↑Atg12	[112]
	OVCA-420, OVCA-429	1.5–500 mg/mL	↓CXCR4, ↓MMP-9	[110]

## Data Availability

Not applicable.

## Abbreviations

OC: Ovarian cancer; SB, *Scutellaria baicalensis*; VEGF, Vascular endothelial growth factor; CDK, cell cycle protein-dependent kinases; PTEN, phosphatase and tensin homolog; EGFR, epidermal growth factor; IKKβ, inhibitory kappa beta kinase β; EMT, epithelial to mesenchymal transition; LGRs, Leucine-rich-repeat-containing G protein-coupled receptors; HMGA2, high mobility group AT-hook 2; ECM, extracellular matrix; MMPs, matrix metalloproteinases; PDTC, pyrrolidine dithiocarbamate; YAP, Yes-associated protein; LC3-II, protein light chain 3-II; ERK, extracellular regulated protein kinases; EZH2, Enhancer of zeste homolog 2; FOXO1, forkhead box protein O1; PPARγ, peroxisome proliferator-activated receptor γ; ALDH1A2, aldehyde dehydrogenase family one member A2; P-gp, P-glycoprotein; CXCR4, C-X-C chemokine receptor type 4.
